# Sonochemical synthesis of nanoparticles from bioactive compounds: advances, challenges, and future perspectives

**DOI:** 10.1016/j.ultsonch.2025.107559

**Published:** 2025-09-10

**Authors:** Gabriela Jajko-Liberka, M.G. Anagha, Paulina Chytrosz-Wróbel, Piotr Kubisiak, Waldemar Kulig, Lukasz Cwiklik, Andrzej Kotarba

**Affiliations:** a Faculty of Chemistry, Jagiellonian University in Krakow, Gronostajowa 2, Krakow 30-387, Poland; b Department of Physics, University of Helsinki, P.O. Box 64, Helsinki FI-00014, Finland; c J. Heyrovský Institute of Physical Chemistry, Czech Academy of Sciences, Dolejškova 3, Prague 18223, Czech Republic

## Abstract

Sonochemical synthesis, driven by acoustic cavitation, has emerged over the past two decades as a powerful and versatile method for producing nanoparticles of bioactive substances with enhanced physicochemical and biological properties. The extreme conditions generated by the collapse of cavitation bubbles – such as transient high temperatures and pressures – facilitate molecular fragmentation, nucleation, and controlled nanoparticle growth. These characteristics make sonochemistry particularly well suited for nanosizing pharmaceuticals, enzymes, and natural bioactive compounds. In addition to a wide range of experimental techniques, recent advances in molecular dynamics simulations have offered critical insights into the molecular-level mechanisms underlying sonochemical processes, revealing the roles of solvent interactions and interfacial dynamics in determining nanoparticle size and stability. This deeper understanding has enabled more precise tuning of synthesis parameters to achieve desired nanoparticle characteristics. Sonochemically synthesized nanoparticles have demonstrated significant potential in diverse biomedical fields, including targeted drug delivery, sonodynamic therapy, regenerative medicine, and antimicrobial coatings. This minireview consolidates two decades of research, highlighting key advancements in the sonochemical synthesis of nanoparticles of bioactive substances, with a focus on molecular-level insights, biomedical applications, future research directions, ongoing challenges, and future perspectives.

## Highlights

• Summarizes two decades of experimental progress in sonochemical synthesis.
• Explores cavitation-driven mechanisms of nanoparticle formation.
• Provides mechanistic insight through molecular dynamics simulations.
• Connects sonochemistry to drug delivery and therapeutic applications.
• Highlights ultrasound-assisted nanosizing for biomedical use.

## Graphical abstract

**Figure:**
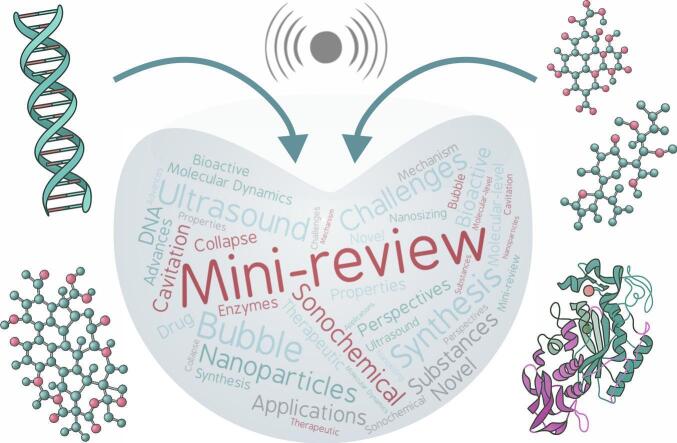

## 1 Introduction

Sonochemistry is a well-established subfield of chemistry that explores the chemical effects induced by ultrasound. Ultrasound is increasingly recognized for its potential to reduce operational costs by minimizing or eliminating ancillary processing steps. This technique enables chemical processes to occur under milder conditions – such as lower temperatures and pressures – reduces reliance on expensive solvents, and streamlines synthetic pathways while enhancing overall product yields. Moreover, it allows for the use of lower-purity reagents or solvents and improves the efficiency of existing catalytic systems. These advantages position ultrasound as a promising and innovative methodology for the efficient production of high-value chemicals and pharmaceuticals [[1], [2], [3], [4]].

**Fig. 1:**
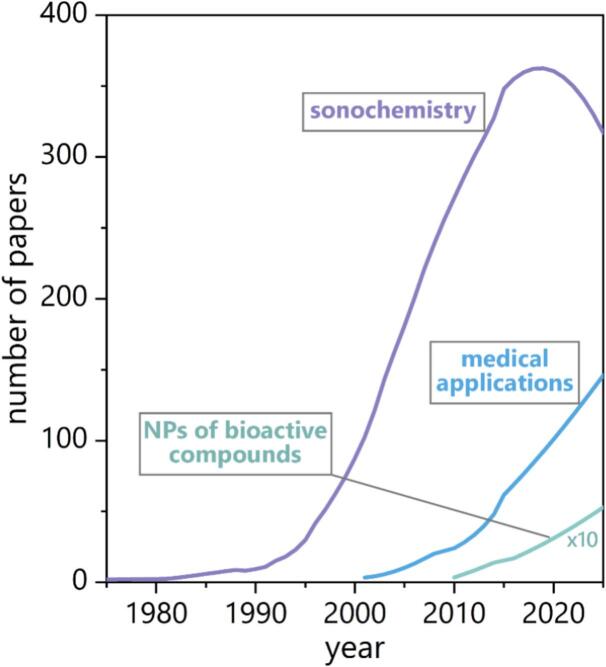
Overview of available literature on sonochemistry. Data Source: Scopus. Keywords: “Sonochemistry,” “Sonochemistry + medical applications,” and “Sonochemistry + bioactive compounds.” Access date: June 23, 2025.

The numerous advantages associated with ultrasound have contributed to a significant increase in publications within the field of sonochemistry over the past decades (Fig. 1). As inferred from publication trends, the subfield focused on nanoparticles of bioactive molecules has emerged over the past decade. Foundational research, primarily conducted by chemists and physicists, has established that the chemical and certain mechanical effects of ultrasound are attributable to the implosive collapse of cavitation bubbles. Despite growing interest from chemical engineers in harnessing and quantifying these effects, the research addressing key applied domains – such as mass transfer, reaction kinetics, reaction modeling, and reactor design – remains limited.

Bioactive nanoparticles play a critical role in modern medicine by offering unprecedented opportunities for targeted drug delivery [5], diagnostic imaging [6], and therapeutic interventions [7,8]. Their physicochemical properties enable precise interactions with biological systems, thereby improving therapeutic efficacy [[9], [10], [11]] and minimizing adverse effects [12,13]. These outcomes are achieved by modulating release kinetics and ensuring controlled and sustained drug concentrations at the target site, such as specific tissues or cells [14].

The overuse and misuse of antibiotics have contributed to the emergence of resistant bacterial strains, rendering once-treatable infections increasingly difficult – or even impossible – to manage [15,16]. As a result, bioactive nanoparticles are being explored for the development of novel antimicrobial agents [17,18]. These nanoparticles can also exhibit high specificity toward targeted cells (e.g., pathogens), thereby minimizing off-target effects while enhancing therapeutic efficacy [19]. Surface engineering of nanoparticles enables them to penetrate biofilms – a common protective mechanism employed by bacteria – making them effective against persistent and antibiotic-resistant infections [[20], [21], [22]].

This paper presents a minireview of the existing literature on the synthesis of nanoparticles of bioactive substances, with the aim of supporting and encouraging future advancements in sonochemical applications.

## 2 Theoretical Background of sonochemical processes

The introduction of ultrasound waves (frequencies above 20 kHz) into a medium generates alternating regions of compression and rarefaction. This phenomenon is common to all types of sound waves, regardless of the medium or physical state. In solutions, the rarefaction phase –corresponding to the negative pressure region of the ultrasound wave – facilitates the formation of cavitation bubbles. This process occurs when the ultrasound intensity is sufficiently high to reach the cavitation threshold. Importantly, this threshold is significantly lower than the pressure required to overcome the cohesive forces between liquid molecules, due to the presence of pre-existing cavitation nuclei in the liquid [23]. These bubbles, typically a few micrometers in diameter, undergo rapid adiabatic collapse in the compression phase within microseconds (10^−6^ s). The processes of bubble formation, growth, and collapse – collectively referred to as acoustic cavitation – constitute the fundamental mechanism underlying the observed effects in sonochemistry [24]. The collapse of an acoustic bubble is accompanied by extreme localized temperatures (∼5,000 K) and pressures (∼1,000 bar). This concept, originally proposed by Noltingk and Neppiras [25], is widely recognized as the “hot spot theory” and remains the most established explanatory model to date [26,27]. Other theoretical frameworks, such as the thermochemical theory [28,29] and Jarman’s mechanothermal theory [30], also incorporate thermal effects to explain the origins of sonochemical reactions and sonoluminescence.

**Fig. 2:**
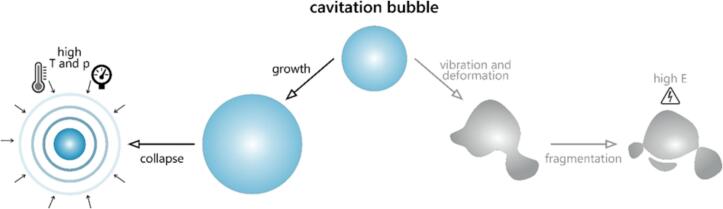
Schematic representation of the fate of a cavitation bubble, highlighting the key physical processes involved [35].

Alternative explanations, including electrical theories, have also been proposed since the earliest investigations into acoustic phenomena. One such model suggests the accumulation of electric charges on opposite sides of the bubble at the moment of its formation [31]. Other hypotheses [32] have also been postulated; however, they have failed to present convincing experimental support [33]. Over time, new theoretical approaches have emerged, incorporating more comprehensive scientific explanations. One such theory proposes that the cavitation bubble undergoes vibrations that lead to its fragmentation. During this process, extremely high local electric fields (>10^11^ Vm^−1^) are generated [34]. Mason and Luche [35] proposed that the actual mechanism may involve a combination of both thermal and electrical phenomena, with the dominant pathway depending on the specific reaction conditions. While the thermal and mechanical effects of acoustic cavitation are widely recognized as the primary explanations for most experimental observations, electrical effects have occasionally been suggested as an alternative. Fig. 2 illustrates both of the proposed pathways in sonochemical reactions.

## 3 Role of sonochemistry in nanoparticle formation

**Fig. 3:**
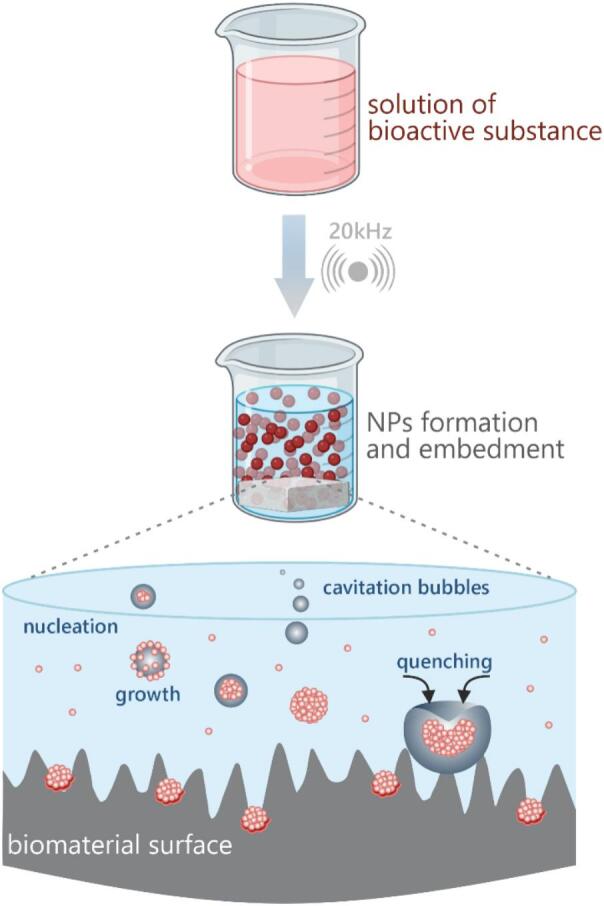
Schematic representation of the sonochemical formation of nanoparticles and their subsequent deposition onto a substrate surface.

Studies by Suslick et al. [36] have demonstrated that the temperature and pressure within cavitation bubble clouds can reach 5,000 K and 1,000 bar, respectively. In isolated bubbles, these parameters may be even higher [[37], [38], [39], [40]]. Because of the aforementioned hot spot mechanism, these extreme conditions drive the formation of highly reactive free radicals through molecular fragmentation. The collapse of a cavitation bubble generates a shock wave that propagates through the surrounding liquid medium. Additionally, when a solid surface is present near the collapse site, liquid microjets can be produced due to the disruption of the bubble’s spherical symmetry. These intense physical and chemical effects can lead to the cleavage of chemical bonds. Primary and secondary sonochemical reactions occur when gas molecules trapped inside the bubble dissociate, forming reactive species. The resulting products are subsequently transported into the surrounding liquid medium. The nature of the solvent strongly influences the physical processes and chemical reactions involved. Numerous studies suggest that these sonochemical effects facilitate the formation of nanoparticles [[41], [42], [43], [44]]. Depending on the conditions, the resulting nanoparticles may be amorphous, as the extremely short-lived cavitation events can lead to rapid cooling that hinders crystallization. However, it is also well established that acoustic cavitation can promote crystal formation – a process widely known as sonocrystallization [45,46]. The complex interplay of chemical and physical phenomena can therefore result in nanoparticles with diverse compositions, and morphologies. In the past two decades, research on ultrasound-assisted nanoparticle synthesis has expanded significantly, revealing its potential in the development of nanostructured bioactive substances (Fig. 1). This approach is particularly compelling given the ability of such substances to interact with biological systems and trigger specific physiological responses. From a biomedical perspective, bioactive substances can stimulate reactions in living tissues [47,48]. These include a wide range of entities, such as small molecules, peptides, drugs, nucleic acids, proteins, and enzymes. The sonochemical synthesis of nanomaterials has attracted considerable attention in recent years. In the context of bioactive nanoparticle synthesis, sonochemistry has proven advantageous in enhancing biocompatibility and functionality, especially for biomedical applications such as drug delivery, imaging, and antimicrobial therapies. By tailoring the precursor solution and reaction environment, nanoparticles can be engineered to incorporate bioactive components – such as plant extracts, peptides, or polymers – either directly during synthesis or through post-synthetic modification (Fig. 3). Furthermore, the scalability, eco-friendliness, and minimal use of toxic reagents in sonochemical processes align well with the principles of green chemistry. These attributes make sonochemistry particularly attractive for producing nanoparticles intended for sensitive applications, such as in healthcare or food packaging, where minimizing environmental and health risks is imperative [49].

## 4 Characterization techniques

Comprehensive characterization of bioactive substances is essential for understanding their physicochemical properties, structural attributes, and functional behaviors, all of which are critical to ensure efficacy and safety across various applications. A range of advanced experimental techniques is employed to achieve this level of insight.

**Fig. 4:**
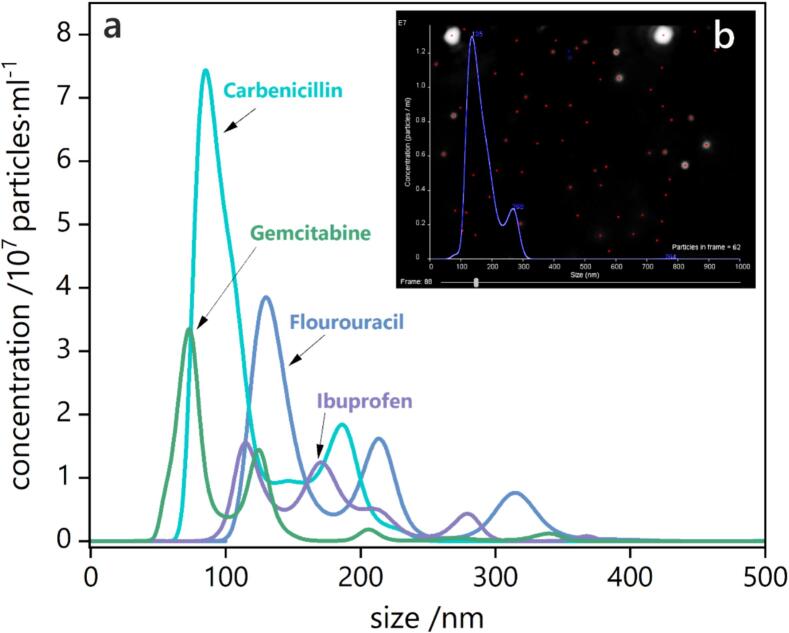
(a) Nanoparticle tracking analysis (NTA) graphs showing particle size versus concentration for various drug formulations evaluated by our research group [51,52]. (b) Representative screenshot of typical nanoparticle tracking visualization using the NTA software.

Nanoparticle tracking analysis (NTA) enables real-time visualization of bioactive nanoparticles. It is a particularly well-suited technique for biomedical applications, as it allows for the analysis of particles in suspension, facilitates live counting and sizing, and detects particles ranging from 10 to 1,000 nm in size. As a multi-parameter analysis method, NTA is both cost-effective and valuable for preliminary analysis prior to the use of more sophisticated microscopic techniques [50]. Representative NTA results for various drug formulations investigated by our team are presented in Fig. 4.

Dynamic Light Scattering (DLS) is another widely used technique for determining the size distribution of nanoparticles in colloidal suspensions. DLS measures fluctuations in the intensity of scattered light caused by the Brownian motion of particles. The technique provides rapid data on particle size and colloidal stability, the latter assessed via zeta potential – an important indicator of dispersibility and long-term stability of bioactive substances [53].

Scanning electron microscopy (SEM) and transmission electron microscopy (TEM) offer high-resolution imaging for analyzing the morphology and structure of bioactive nanoparticles at nanoscale. SEM provides detailed information on surface topology and particle morphology, while TEM reveals internal structures, crystallinity, and interparticle arrangements. Such imaging is essential in applications where surface characteristics and internal architecture influence functionality [54]. Representative SEM and TEM images of bioactive nanoparticles synthesized via sonochemical methods are presented in Table 1.

**Table 1:** Summary of bioactive substances synthesized via sonochemistry, including the solvent used, sonication parameters, nanoparticle size, and representative images.

Solvent/Parameters (amplitude, power, time)	Size (nm)	Figure	Refs
Drugs			
Tetracycline			
Water/dodecane: 22 %, 750 W, 1–10 min	50	See Fig. 6a	[59]
Water: 35 %, 5–20 min	21	See Fig. 7a	[60]
Water: 22 %, 0.5–30 min	15–20		[57]

Curcumin			
Water: 500 W, 20 min	170–230		[61]
Water: 120–300 W, 6–12 s	50–800		[62]
Ethanol, methanol, acetone, and ethyl acetate: 130–250 W, 60 min	5 × 10^4^ (after extraction)		[63]

Fluorouracil			
Water/ethanol: 35 %, 5 min	80–120		[51]

Gentamicin			
Water/ethanol: 30 %, 6 min	30–70		[64]

Tannic acid			
Water: 30 %, 750 W, 60 min	40		[65]

Penicillin			
Water: 25 %, 750 W, 10 min	70		[66]*

Enzymes			
Pepsin			
Potassium acetate: 25 %, 750 W, 30 min:	80–140		[67]
α-amylase			
Water: 25 %, 750 W, 30 min	30–700	−	[68]
Water: 25 %, 750 W, 30 min	40–150		[69]

Cellobiose dehydrogenase			
−: 22 %, 750 W, 3–30 min	65–93		[70]

Vitamins			
Vitamin B12			
Water: 25 %, 750 W, 10 min	120–180	See Fig. 6b	[66]
Nanospheres			
DNA			
Water/dodecane oil or soya oil: 39–40 %, 26.7 Wcm^−2^, 3 min	260	See Fig. 7b	[71]

Carbohydrates			
Starch			
Water: 154 W, 15 min	80		[72]
Water: 100 W, 30 min	77		[58]
Water: 100 W, 30 min	453		[73]
Water: 30 min	615		[74]

Carboxymethylcellulose			
Phosphate buffer solution: 500 Wcm^−2^, 8 min	200–300		[75]
Nanoemulsions/nanocarriers			

Anise extract			
Water with 5 % Tween 80: 50 %, 3 min	400		[76]

Oil in Alginate-Chitosan Nanoparticles			
Water and surfactant: 3 min	320–340		[77]

Eugenol-loaded chitosan			
Acetic acid with Tween 20: 750 W, 0–15 min	100		[78]

Vitamin C-loaded, chitosan-coated nanoliposomes			
Ethanol: 20 W, 2 min	96 – 97		[79]

*International Journal of Nanomedicine 2015:10 3593–3601. Originally published by and used with permission from Dove Medical Press Ltd.

**Fig. 5:**
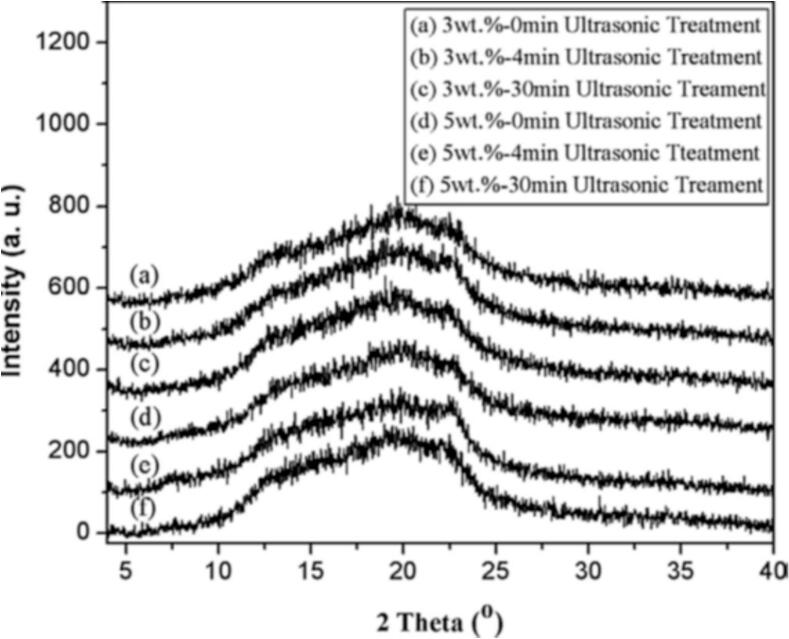
X-ray diffraction patterns of starch-derived nanoparticles synthesized from starch pastes with varying concentrations and ultrasonic treatment durations. The figure and legend are reused with permission [58].

Spectroscopic methods are also essential for characterizing the molecular structure, functional groups, and interactions of bioactive materials. Fourier transform infrared spectroscopy (FTIR), for instance, identifies specific functional groups and confirms surface modifications or coatings [55]. The crystalline structure, phase composition, and degree of crystallinity of bioactive substances are equally important and can be determined using X-ray diffraction (XRD). This technique confirms the formation of desired phases, their crystallinity, and molecular arrangement, and ensures the absence of unwanted impurities [56]. Notably, ultrasonic irradiation generally does not disturb the structural integrity of bioactive substances [51,57], making XRD a reliable method for validating synthesis protocols, particularly in sensitive applications such as pharmaceuticals or the food industry. Fig. 5 compares XRD patterns of starch nanoparticles synthesized with and without ultrasonic treatment.

Proper characterization is vital for the practical deployment of bioactive substances. In nanomedicine, inadequate characterization can lead to batch-to-batch variability, reduced efficacy, or even toxicity. In environmental applications, poorly characterized nanoparticles may behave unpredictably, raising ecological concerns. Therefore, advanced characterization techniques are indispensable for ensuring that bioactive substances meet stringent standards of safety, efficacy, and regulatory compliance.

## 5 Application of sonochemistry in the synthesis of nanoparticles of bioactive substances

Fig. 1 illustrates that the use of sonochemistry for synthesizing nanoparticles of bioactive molecules dates back to the early 21st century. Since then, numerous studies have employed this approach, with some utilizing it as a one-step process to both synthesize nanoparticles and embed them onto various substrates. The widespread adoption of this technique is likely due to its high success rate and efficiency in producing well-defined nanoparticles with controlled properties. Table 1 summarizes key studies on the sonochemical synthesis of various bioactive substances, including the type of solvent, sonication parameters, resulting nanoparticle sizes, and representative images.

## 6 Drugs

**Fig. 6:**
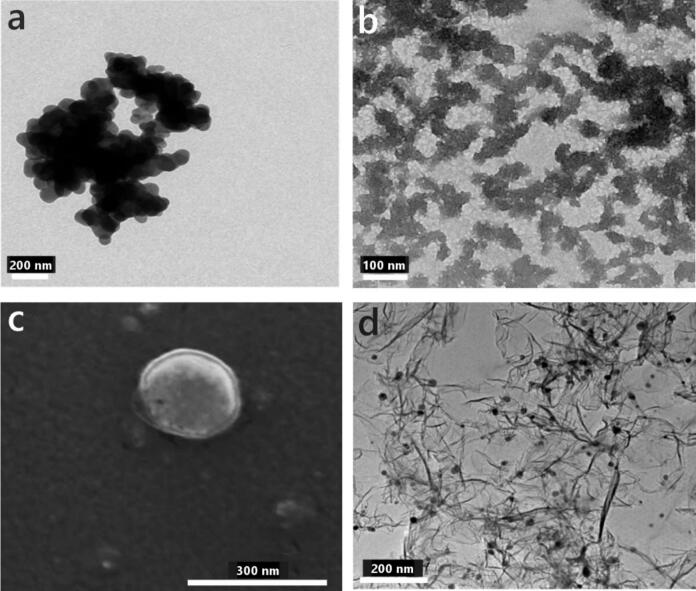
(a) Transmission electron microscopy (TEM) image of tetracycline (TET) nanoparticles obtained in solution following sonication. (b) TEM image of vitamin B12 nanoparticles after 5 min of ultrasonic radiation. (c) Scanning electron microscopy image of a 280-nm double-stranded DNA (dsDNA) nanosphere that was produced from genomic DNA extracted from cells. (d) TEM image of TET nanoparticles deposited on graphene oxide sheets, showing size variation as a function of 5-min ultrasonication time. The figures and legends are reproduced from the following references with permission: (a) [59]; (b) [66] (International Journal of Nanomedicine 2015:10 3593–3601; originally published by and used with permission from Dove Medical Press Ltd.); (c) [71]; (d) [60].

Tetracyclines (TETs) are classified as broad-spectrum antibiotics and are widely used against various pathogens, including bacteria, parasites, and mycoplasmas [80]. Despite their widespread application, limited research has been published on their synthesis into nanoparticles via sonochemistry. Gedanken et al. [59] reported the synthesis and subsequent embedment of TET nanoparticles into a Parylene C polymer (poly(chloro-para-xylylene)), noting that particle size and surface deposition were highly influenced by sonication duration, precursor concentration, and the type of organic solvent. An optimal medium concentration yielded the best results. In another study, the same group deposited TET nanoparticles onto graphene oxide (GO) sheets using ultrasound [60]. They observed that nanoparticle size increased with sonication time. Fig. 6d shows the variation in nanoparticle size on GO sheets as determined using TEM. Shimanovich et al. [57] further demonstrated that brief ultrasound exposure produced larger nanoparticles, while prolonged sonication yielded smaller ones. However, excessive exposure led to the disappearance of nanoparticles. Notably, the smallest nanoparticles were formed under an argon atmosphere, which was attributed to the formation of smaller cavitation bubbles.

Zabihi et al. [62] explored a modified supercritical antisolvent process involving ultrasound vibration to encapsulate curcumin nanoparticles in poly(lactic-co-glycolic acid) (PLGA). Increasing the ultrasonic power to 240 W resulted in smaller and more uniform particles, whereas prolonged static agitation produced larger ones. Similarly, Shirsath et al. [63] reported that 20 min of ultrasound exposure led to the formation of curcumin nanoparticles ranging in size from 170 to 230 nm using a liquid antisolvent precipitation method.

In one of our studies [64], we successfully synthesized gentamicin nanoparticles and embedded them onto a Parylene C surface using a one-step method. The approach involved pretreating the substrate with oxygen plasma in order to create nanotopography for effective nanoparticle entrapment. We emphasized the importance of optimizing ultrasound parameters to ensure a suitable nanoparticle size for drug delivery. In another study [51], we synthesized fluorouracil nanoparticles – an anticancer drug – using ultrasound. It was reported that the water/ethanol solvent interface significantly influenced nanoparticle nucleation.

Perelshtein et al. in 2014 [65] reported the synthesis of tannic acid NPs and their simultaneous immobilization onto cotton fabric using ultrasound. Nanoparticles ∼ 40 nm in size were obtained under optimized conditions. The authors emphasized that nanoparticle embedment or coating occurred only in the presence of ultrasound, as attempts without it did not yield favorable results.

Yariv, Gedanken, and collaborators [66] reported enhanced biological activity of penicillin and vitamin B12 following sonochemical nanoparticle synthesis. Although ultrasound conditions remained constant, varying concentrations were used. Nanoparticle size decreased with increased sonication time. Fig. 6b shows vitamin B12 NPs formed after 5 min of ultrasound exposure.

In addition, sonochemistry has been employed for the nanosizing of other antibiotics, including ciprofloxacin and amoxicillin [[81], [82], [83], [84]]. These processes typically involve ultrasonic irradiation of precursor solutions containing the drug and stabilizing agents (e.g., surfactants or polymers) under carefully controlled conditions of frequency, power, and temperature. Cavitation-induced microenvironments facilitate rapid nucleation and growth of nanoparticles, often yielding smaller and more uniform systems compared to conventional techniques. For instance, ciprofloxacin-loaded nanoparticles were synthesized using high-intensity ultrasound at 20 kHz and moderate power levels, resulting in particles with enhanced dispersibility and controlled release profiles. Similarly, ultrasound-assisted encapsulation of amoxicillin into polymeric carriers such as PLGA has been reported, where optimization of sonication power and duration was critical to achieve reduced particle size while maintaining drug stability. Compared to traditional formulations, these sonochemically synthesized antibiotic nanoparticles exhibited improved bioavailability, reduced burst release, and greater suitability for sustained therapeutic delivery.

## 7 Enzymes and DNA

**Fig. 7:**
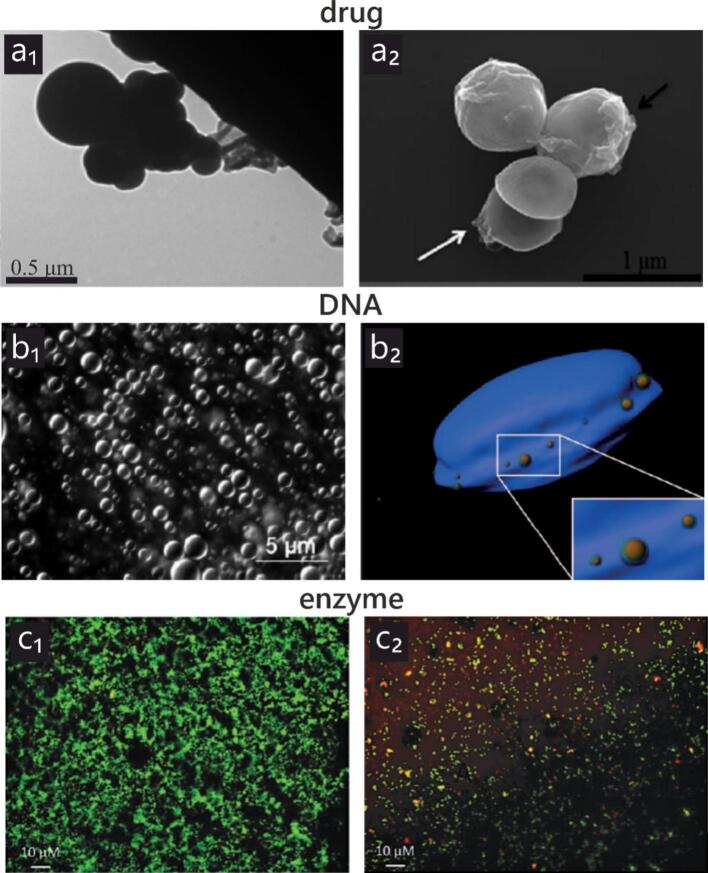
(a1) TEM image of a sonochemically synthesized TET nanoparticle. (a2) TEM image of GO/TET composites interacting with *Staphylococcus aureus*. (b) Light microscopy image of dsDNA nanospheres produced from genomic DNA extracted from cells, along with the corresponding three-dimensional image showing the distribution of DNA nanospheres within human U2OS cancer cells. (c) Confocal laser scanning microscopy images of LIVE/DEAD-stained *S. aureus* colonies attached to untreated polydimethylsiloxane sheets and to corresponding cellobiose dehydrogenase-immobilized sheets. The figures and legends are reproduced from the following references with permission: [60,70,71].

Numerous studies have investigated the immobilization of enzyme nanoparticles onto various substrates using high-intensity ultrasound. This approach is motivated by the fact that immobilization not only enhances enzyme stability but also facilitates their recovery and reuse, which is highly advantageous for industrial and biomedical applications. Sonochemistry provides an efficient, clean, and versatile route for immobilization without the need for harsh chemical reagents, making it a greener alternative to conventional immobilization methods. Moreover, ultrasound enables fine control over particle size, morphology, and distribution, which is often difficult to achieve with purely chemical techniques. Meridor and Gedanken synthesized α-amylase nanoparticles and embedded them into various substrates [68,69]. They observed a strong correlation between initial enzyme concentration and particle size: lower concentrations produced smaller nanoparticles. A similar trend was observed for pepsin nanoparticles [67]. Lipovsky et al. [70] confirmed this concentration-dependent effect in their study on cellobiose dehydrogenase (CDH) nanoparticles. They also found that shorter sonication times at higher enzyme concentrations resulted in smaller particles. Fig. 7c illustrates the sonochemically formed CDH NPs.

Ultrasound has been well established as an effective technique for DNA fragmentation, both with and without the involvement of cavitation effects [85,86]. Shimanovich et al. [71] employed sonochemistry to synthesize chemically stable DNA nanoparticles from double-stranded DNA (dsDNA). This top-down approach demonstrates the potential of sonochemistry as a powerful method for converting DNA from various sources into nanomaterials in aqueous media. Fig. 7b presents SEM and light microscopy images of dsDNA nanospheres. However, the conversion efficiency was shown to depend on the surrounding atmosphere, with higher yields observed under argon. Importantly, the biological activity of the synthesized nanospheres remained intact following ultrasound irradiation.

It is important to note, however, that enzymes and nucleic acids are highly sensitive biomolecules that can be adversely affected by the extreme physical and chemical conditions generated during cavitation. The transient hotspots may cause denaturation of proteins, loss of enzymatic activity, or cleavage of nucleic acids if the process is not carefully controlled [87]. The underlying reason is that the biological activity of enzymes and DNA depends on complex structural organization stabilized by relatively weak interactions. In proteins, proper folding relies on hydrogen bonds, hydrophobic interactions, and – in some cases – disulfide bridges (–S–S–). Such non-covalent forces are highly vulnerable to thermal fluctuations, mechanical stress, and oxidative attack. In nucleic acids, the double helix is stabilized primarily by hydrogen bonds between complementary bases and π–π stacking interactions. These are also easily disrupted by rapid heating–cooling cycles or mechanical shear, while reactive oxygen species can induce strand breaks or oxidative lesions in the sugar–phosphate backbone and bases. In contrast, small molecules such as drugs or simple metabolites, which are held together predominantly by stronger covalent bonds and lack complex tertiary structures, are generally more resistant to transient cavitation conditions. Therefore, larger biomolecules like enzymes and DNA are inherently more prone to structural damage under high-energy ultrasound exposure, which highlights the importance of carefully optimizing sonication parameters to balance nanoparticle formation with biomolecular stability.

Nevertheless, several studies have demonstrated that by optimizing ultrasonic parameters – including frequency, power density, pulse mode, and irradiation time – it is possible to minimize these detrimental effects while taking advantage of sonochemical conditions to achieve nanoparticle formation. For enzymes, shorter irradiation times at moderate powers can promote nanoparticle formation while maintaining catalytic activity. In the case of DNA, controlling the gaseous atmosphere (e.g., using argon) can reduce oxidative damage, thereby improving yields and preserving structural integrity. This tunability is one of the main advantages of sonochemistry: it offers a controllable, reproducible, and environmentally friendly method for preparing functional biomolecular nanomaterials.

## 8 Carbohydrates

Starch nanoparticles (SNPs) have gained significant attention across various fields – including food, pharmaceuticals, and cosmetics – due to their unique properties compared to native starch, especially when synthesized using ultrasound. In a study by Chang et al. [58], an aqueous paste of potato starch was used instead of a true solution, as suggested in earlier research. The authors employed an on/off ultrasound pulse sequence of 2 s/4 s to prevent overheating of the sample. A significant reduction in the apparent viscosity of the starch paste was observed during ultrasound treatment, with the most notable decrease occurring within the first 15 min of exposure. This reduction in viscosity was attributed to the cessation of chain scission once a minimum chain length was reached [88,89]. Moreover, higher precursor concentrations yielded larger nanoparticles, while ultrasound treatment resulted in a more uniform size distribution, as indicated by a lower polydispersity index.

Shabana et al. in 2019 [72] reported on ultrasound-assisted acid hydrolysis for the synthesis and structural modification of potato SNPs. The authors also explored the coating of SNPs with ascorbic and oxalic acids, targeting applications in the food industry. A 3 s/1 s on/off pulse sequence was employed during nanoparticle synthesis. The results indicated a reduction in the particle size of native starch from 1,596 nm to 80 nm after ultrasound treatment. In a similar study, Gonçalves et al. [73] examined the modification of native starch from *Araucaria angustifolia* using ultrasound and acid hydrolysis. While acid hydrolysis produced smaller nanoparticles, ultrasound was found to be effective in structurally modifying the starch. The experimental protocol involved a one-min pause following each one-min ultrasound cycle. The significance of pulse sequences in SNP synthesis is consistently emphasized in the literature. Ahmad et al. [74] further supported this in their studies on SNPs and the nanoencapsulation of catechin. In this study, SNPs were synthesized from horse and water chestnuts as well as lotus stems, using ultrasound. A 30-min ultrasound treatment was applied with 5-min intervals to prevent overheating of the aqueous starch solution. For catechin encapsulation, catechin hydrate in ethanol was added prior to sonication.

A growing body of literature also highlights ultrasound-assisted synthesis of nanocarriers, particularly in food industry–driven research. These include lipid-based nanocarriers [90,91] and those composed of bio- and synthetic polymers [92]. While a detailed description of these investigations is beyond the scope of this mini-review, Table 1 includes selected examples to illustrate the efficacy of the technique and the influence of sonication parameters. For instance, Ghazy et al. [76] reported the preparation of nanoemulsions from anise seed extract using ultrasound to enhance antibacterial activity. The required radiation exposure time for synthesis was only 3 min, yielding nanoemulsions with an average size of 400 nm. Similarly, Elgegren et al. [77] synthesized alginate particles in oils (sacha inchi, soybean, and olive), with an average size of ∼ 340 nm, using a 3-min ultrasound treatment.

## 9 Enhanced bioactivity of sonochemically synthesized nanoparticles

Several of the abovementioned studies not only successfully synthesized nanoparticles via sonochemistry but also demonstrated improved bioactivity of the resulting materials. Nanosizing has been shown to enhance the pharmacological efficiency of various substances compared to their bulk counterparts. For example, in the case of sonochemically prepared GO/TET composites, bacterial cell walls were perforated due to complete wrapping or engulfing by the composite. The TEM images in panel *a* of Fig. 7 illustrate the antibacterial activity of the drug nanoparticles. Gedanken et al. [59] also demonstrated that TET-coated Parylene C surfaces exhibited strong antibacterial resistance, citing sonication as a simple and effective method for developing antimicrobial surfaces. Yariv et al. [66,93] attributed the enhanced efficacy of penicillin and vitamin B12 to increased surface area and nanocrystal formation following sonication. Fig. 7b shows a light microscopy image of synthesized DNA nanospheres and the corresponding three-dimensional image of their distribution on human U2OS cancer cells. Shimanovich et al. [71] also investigated the ability of DNA nanospheres to enter bacterial cells, suggesting their potential as delivery agents of genetic material to cells. Similarly, in another study, Lipovsky et al. [70] examined CDH deposition on polydimethylsiloxane (PDMS) polymer sheets and tested their antibacterial activity against *Staphylococcus aureus*. Fig. 7c1 and c2 present the confocal laser scanning microscopy images of LIVE/DEAD-stained bacterial colonies on the polymer surface. The enzymatic nanoparticles not only exhibited antimicrobial effects but also reduced bacterial adhesion. However, this effect was found to be strongly dependent on sonication time.

## 10 Sonication parameters

While sonochemistry offers numerous advantages for nanoparticle synthesis, achieving reproducibility across different systems requires careful optimization of sonication parameters. Key variables such as power intensity, ultrasound frequency, and exposure time significantly influence the physicochemical characteristics of the resulting nanoparticles [94]. Understanding the role of each parameter is essential for tailoring nanoparticles to specific applications.

### 10.1 Effect of power

**Fig. 8:**
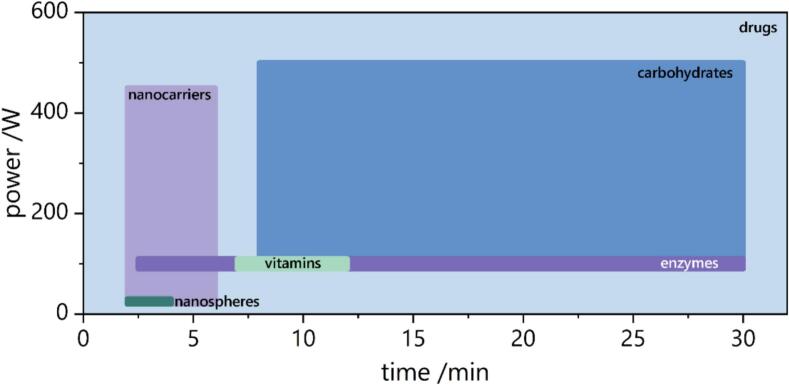
Influence of sonication power and duration on the synthesis of various classes of bioactive substances.

Ultrasound power directly affects the intensity of cavitation events. Higher power levels generally promote more vigorous bubble dynamics, often resulting in locally elevated temperatures and pressures. This can enhance the fragmentation of precursor molecules and promote the formation of smaller, more uniform nanoparticles. For example, in the sonochemical synthesis of gold nanoparticles, increasing the power from 60 W to 210 W resulted in a decrease in particle size from 16 nm to 12 nm [95]. However, at relatively low ultrasonic frequencies, excessive acoustic power can lead to a decrease in bubble collapse temperatures [96]. Moreover, excessive power may also cause overheating, degradation of sensitive bioactive compounds, or even the destruction of already-formed nanoparticles. Therefore, identifying an optimal power threshold is critical for balancing efficiency with nanoparticle stability (see Fig. 8).

### 10.2 Effect of frequency

Ultrasound frequency, which defines the number of acoustic cycles per second, influences both the size and behavior of cavitation bubbles. Lower frequencies (e.g., <100 kHz) produce larger bubbles and more intense collapses, favoring mechanical effects such as particle fragmentation. In contrast, higher frequencies (e.g., >100 kHz) generate smaller bubbles and result in more uniform energy distribution, which may be better suited for delicate molecular systems or controlled chemical reactions. For example, in the sonochemical synthesis of platinum nanoparticles, a frequency of 408 kHz resulted in higher sonochemical efficiency and smaller particle size (2.3 nm) compared to 20 kHz, which yielded particles of 2.7 nm [97]. The selected frequency must align with the desired nanoparticle characteristics and the sensitivity of the bioactive compound.

### 10.3 Effect of time

Sonication time determines the duration of exposure to cavitation effects. Shorter durations may result in incomplete nanoparticle formation or inadequate surface functionalization, while prolonged exposure can cause particle agglomeration, degradation, or a reduction in bioactivity. Optimizing sonication time is particularly critical when working with bioactive compounds, as preserving molecular integrity is essential. Research has shown that nanoparticle size generally decreases with increasing sonication time up to a certain threshold, beyond which further exposure may reverse this trend or damage the final product. For example, a study by Dong et al. [98] showed that egg-white protein–curcumin nanoparticle assembles under varying sonication durations. Extending sonication to 12 min enhanced protein solubility, surface hydrophobicity, and encapsulation efficiency. However, with longer, excessive sonication, SEM revealed blocky aggregates and increased crystallinity – signaling destabilization and reduced quality of the nanocomplex.

Recent advances also highlight the use of cavitation agents as a promising strategy to improve energy efficiency and reaction control in sonochemistry. These agents, typically solid particles with engineered surface properties, enhance cavitation activity by promoting localized bubble nucleation and collapse near solid–liquid interfaces. Such spatially selective cavitation enables more efficient generation of reactive species and facilitates targeted transformations of bioactive molecules. For instance, CuO microparticles with multicavity (highly porous) architecture have been reported to significantly increase hydroxyl radical production while reducing overall energy input [94,99]. Importantly, these strategies are well aligned with the principles of sustainable nanoparticle synthesis, as they provide improved control over particle size and morphology while minimizing potential degradation of sensitive bioactive compounds. Although still at an early stage, the integration of cavitation agents into sonochemical systems represents a particularly promising direction for future research in the fabrication of nanoparticles from bioactive compounds.

## 11 Advantages of sonochemical synthesis over traditional methods

Sonochemical synthesis has emerged as a superior method for producing nanoparticles compared to traditional techniques, particularly in terms of yield, particle size, and bioavailability. The localized high-energy conditions generated by acoustic cavitation – reaching temperatures of up to 5,000 K and pressures exceeding 1,000 atm – enable rapid and efficient reactions. These extreme conditions facilitate high nanoparticle yields with minimal by-products, even within short reaction times. In contrast, traditional methods, such as chemical reduction or hydrothermal synthesis, often require prolonged heating or additional energy input, which can reduce overall efficiency and increase the risk of side reactions or incomplete precursor conversion [100,101].

In terms of particle size, sonochemical synthesis allows for precise control, typically producing nanoparticles in the range of 1–100 nm with narrow size distributions. The rapid cooling rates and localized reaction zones prevent excessive particle growth and aggregation. In comparison, traditional methods often suffer from slower reaction kinetics and uneven energy distribution, leading to larger and more variable particle sizes [102]. Such uniformity in particle size is critical for applications such as catalysis, drug delivery, and imaging, where size-dependent properties directly influence performance.

Bioavailability is another key advantage of sonochemical synthesis. Sonochemical methods enable the *in situ* incorporation of bioactive molecules – such as peptides, proteins, or plant extracts – into nanoparticles during synthesis. This enhances solubility, stability, and biological interaction, leading to improved bioavailability. In traditional approaches, functionalization is typically a separate post-synthesis step, which can compromise bioactivity or result in inefficient binding. For example, sonochemically synthesized iron oxide nanoparticles functionalized with folate and cisplatin have shown enhanced anticancer activity, demonstrating the biomedical potential of this approach [103].

The combination of high yield, precise size control, and enhanced bioavailability makes sonochemical synthesis an efficient and versatile approach for nanoparticle production. These advantages have positioned it as a preferred method for developing bioactive nanoparticles for use in medicine, environmental science, and advanced materials engineering [104]. The effectiveness of sonochemical synthesis is closely tied to the design and configuration of the sonochemical reactor. These reactors are engineered to optimize the delivery of ultrasonic energy to the reaction medium, ensuring uniform cavitation and efficient energy transfer.

There are several types of sonochemical reactors [105], including ultrasonic baths, ultrasonic horns, flow reactors, and multifrequency or dual-mode reactors. Ultrasonic baths, commonly used in laboratory settings, provide a uniform but relatively low-intensity ultrasound field. They are suitable for small-scale synthesis but may lack the power needed for more demanding reactions. Ultrasonic horns deliver high-intensity ultrasound directly into the reaction medium, making them ideal for precise and scalable nanoparticle synthesis. These devices allow control over parameters such as amplitude, frequency, and pulse duration. Flow reactors are designed for continuous processing and are increasingly employed in industrial-scale production. They offer improved temperature control, reproducibility, and scalability. Multifrequency and dual-mode reactors are more advanced systems that combine different ultrasound frequencies or integrate ultrasound with other energy sources, such as UV irradiation, to enhance reaction efficiency and selectivity. Recent developments in microfluidic sonoreactors offer a compelling alternative to conventional sonochemical setups, especially for the synthesis of bioactive compounds. Due to their microscale channel dimensions, these reactors provide precise control over reaction conditions, enhanced heat and mass transfer, and improved reproducibility, overcoming several limitations of traditional sonoreactors. Their ability to operate with minimal reagent volumes and safely handle unstable or hazardous reactants makes them especially attractive for pharmaceutical and biomedical applications. As demonstrated in a recent comprehensive study [94], the integration of ultrasound with microfluidic platforms enables process intensification and opens new opportunities for scalable, efficient fabrication of nanoparticles from bioactive molecules.

The development of sonochemical reactors tailored to specific materials and applications is a growing area of research. Key factors considered in reactor design include ensuring the even distribution of cavitation zones to avoid hot spots or inactive regions, preventing overheating – particularly for thermally sensitive bioactive compounds – and designing systems that can be scaled from laboratory to industrial production without compromising product quality. These considerations aim to improve the reproducibility, efficiency, and environmental sustainability of sonochemical synthesis.

## 12 Molecular mechanism based on molecular dynamics (MD) simulations

The theoretical foundations describing cavitation phenomena from physical and macroscopic perspectives are well established [106,107]. Models based on hydrodynamics, thermodynamics, and bubble dynamics can accurately predict bubble oscillation, growth, collapse, and the extreme conditions generated within collapsing bubbles. However, when it comes to the molecular-level description of nanoparticle formation during sonochemical processes, direct simulations remain scarce [108,109]. The lack of atomistic simulations creates a gap between macroscopic theories and the molecular processes underlying nucleation and nanoparticle growth. Although several hypotheses have been proposed, they often lack validation at the molecular level.

Recognizing the challenge of simulating fully atomistic cavitation processes, early computational studies approximated the bubble–solution interface by simulating highly concentrated bulk solutions [110,111]. In these simulations, it was assumed that bioactive molecules accumulate at the bubble interface, leading to a local increase in concentration that promotes aggregation and nanoparticle formation. While informative, these approaches omitted critical factors such as molecular orientation and interfacial curvature, which may significantly influence the nucleation process.

**Fig. 9:**
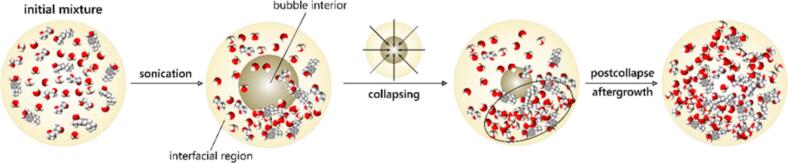
Schematic representation of fluorouracil nanoparticle formation under sonochemical conditions using MD simulations. Upon sonication, cavitation bubbles form within the liquid medium. Molecules accumulate at the bubble–solution interface, leading to localized concentration and specific orientation. During bubble collapse, further concentration and molecular alignment occur, promoting the formation of initial clusters. Following collapse, nanoparticle growth continues, ultimately yielding stable bioactive nanoparticles.

In our recent work [51], we explicitly included the bubble–solution interface in atomistic MD simulations to study fluorouracil nanoparticle formation. These simulations revealed that the interface not only concentrated the molecules but also induced specific molecular orientations (see Fig. 9). Such oriented arrangements promoted the formation of initial clusters and pre-nanocrystalline seeds, which are crucial for subsequent nanoparticle growth. These findings offer molecular-level validation for hypotheses that were previously proposed without direct atomistic evidence.

Importantly, subsequent studies [52] demonstrated that the role of the interface is highly molecule-dependent. For example, in the case of ibuprofen, simulations revealed that nanoparticle formation was governed primarily by solvent–drug interactions, with minimal influence from bubble interfaces. In this case, ethanol strongly stabilized isolated molecules and inhibited aggregation, unlike water. Therefore, for ibuprofen, bubble collapse and cavitation effects appear largely irrelevant to nanoparticle formation.

Other studies further emphasize the importance of solvent effects and interfacial phenomena in nanoparticle formation [[112], [113], [114], [115], [116], [117], [118], [119], [120]]. For example, Bhangu et al. [113] demonstrated metastable assembly driven by cavitation, while Yao et al. [120] highlighted the role of partial melting during nanoparticle collisions. These findings reinforce the understanding that both solvent conditions and interfacial properties play a critical role in determining the pathways for nanoscale aggregation and crystallization.

Given the observed molecular specificity, a tiered simulation strategy is recommended for future investigations. First, simulate bulk solutions at high concentrations. Second, simulate simplified vacuum-liquid interfaces to assess potential interfacial concentration and orientation effects. Third, if significant enhancement at the vacuum-liquid interface is detected, proceed to explicit bubble interface simulations for comprehensive characterization. Technically, bulk solution simulations can be carried out using standard periodic MD boxes, while flat interfaces can be modeled with a periodic slab geometry. To capture the bubble interface and collapse, a constrained vacuum-sphere within a water box setup can be employed, as devised in our previous work [51]. This approach enables efficient identification of systems where interfacial effects are critical, as opposed to those dominated by bulk solvent interactions, thereby optimizing computational resources and informing experimental design.

It should be mentioned that in some aspects of sonochemical nanoparticle formation, classical MD simulations can be assisted by electronic structure calculations, in particular using density functional theory (DFT). While direct electronic-structure simulations of cavitation events are not feasible, DFT provides a powerful complement by probing molecular interactions and properties of the nanoparticles once they form. For example, DFT has been used to analyze the structure and electronic properties of ultrasound-assisted nanomaterials such as SnS_2_ nanoflowers [121], coordination polymers [122], and Cu- or Mn-based complexes [123,124], thereby rationalizing their growth and stability. These studies show that while MD captures the dynamic concentration and orientation effects near bubble interfaces, DFT refines our understanding of the electronic and chemical factors relevant for nanoparticle formation and stability. On a more technical note, DFT can also be used for the initial assessment of molecular geometries and charge distributions of bioactive molecules. This information is valuable for the derivation and benchmarking of empirical force field parameters employed in MD simulations.

Bridging the gap between macroscopic cavitation physics and molecular-level mechanisms of nanoparticle formation is increasingly achievable through advances in MD simulations. Explicit modeling of bubble interfaces has revealed critical phenomena such as molecular enrichment and orientation that contribute to nanoparticle nucleation in certain bioactive compounds. However, as demonstrated, these effects are highly molecule-dependent, emphasizing the need for careful, molecule-specific modeling strategies to advance sonochemical nanomaterials engineering.

## 13 Challenges and future directions

Despite the rapid progress and clear advantages of sonochemical synthesis, several challenges and limitations must still be addressed to fully harness its potential in the fabrication of bioactive nanoparticles. One of the primary issues is the absence of standardized protocols. Variations in ultrasound frequency, power, solvent systems, and precursor concentrations can lead to inconsistent results across different laboratories. While sonochemical synthesis is well established at the laboratory scale, scaling these processes to industrial production remains difficult. Challenges such as reactor design and the development of continuous processing systems must be resolved for successful large-scale implementation. The sonochemical behavior of bioactive molecules is highly dependent on their chemical structure and the surrounding solvent. This molecular specificity necessitates customized optimization for each system, which can be time consuming and labor intensive.

Although recent advances in molecular dynamics simulations have begun to bridge the gap between macroscopic observations and molecular-level understanding, the precise mechanisms of nanoparticle nucleation and growth under sonochemical conditions remain incompletely understood for many systems. Additionally, sensitive bioactive compounds may degrade under intense cavitation conditions, limiting the applicability of sonochemistry to certain classes of molecules.

To address these limitations and enhance the potential of sonochemical synthesis, future research should focus on optimizing synthesis protocols for specific classes of bioactive molecules, broadening the application of molecular simulations to inform experimental conditions for nanoparticle formation, developing scalable reactor systems that maintain cavitation efficiency while enabling continuous or semi-continuous production, and exploring biomedical applications – including targeted drug delivery, gene therapy, antibacterial coatings, and tissue engineering scaffolds – where sonochemically fabricated nanoparticles may offer advantages in terms of biocompatibility and functionality.

## 14 Conclusions

Sonochemical synthesis has emerged as an effective and versatile method for fabricating nanoparticles from bioactive substances. Its unique ability to generate extreme localized physicochemical conditions enables rapid, efficient, and environmentally friendly synthesis, allowing for precise control over particle composition and size. Compared to conventional approaches, sonochemistry enhances bioavailability, functionalization potential, and scalability across diverse applications. Recent advances in molecular dynamics simulations have provided valuable molecular-level insights into the mechanisms of nanoparticle formation, further reinforcing the foundation for future innovation. Nevertheless, challenges related to reproducibility, scalability, and mechanistic understanding remain. Addressing these issues through interdisciplinary research will be essential to fully realize the potential of sonochemistry in nanomedicine, environmental science, and other advanced technological fields.

## CRediT authorship contribution statement

**Gabriela Jajko-Liberka:** Writing – original draft, Visualization, Methodology, Investigation, Data curation, Conceptualization. **Anagha M.G.:** Writing – original draft, Conceptualization, Methodology, Investigation, Data curation. **Paulina Chytrosz-Wróbel:** Writing – original draft, Investigation. **Piotr Kubisiak:** Writing – original draft, Investigation. **Waldemar Kulig:** Writing – original draft, Validation, Investigation, Funding acquisition, Conceptualization. **Lukasz Cwiklik:** Writing – original draft, Validation, Investigation, Conceptualization. **Andrzej Kotarba:** Writing – review & editing, Supervision, Project administration, Funding acquisition, Conceptualization.

## Declaration of competing interest

The authors declare that they have no known competing financial interests or personal relationships that could have appeared to influence the work reported in this paper.
